# Co-occurring trauma- and stressor-related and substance-related disorders in youth: A narrative review

**DOI:** 10.18103/mra.v12i8.5688

**Published:** 2024-08-31

**Authors:** Jesse D. Hinckley, Zachary W. Adams, Trey V. Dellucci, Steven Berkowitz

**Affiliations:** 1 Department of Psychiatry, University of Colorado School of Medicine; 2 Department of Psychiatry, Indiana University School of Medicine; 3 START Center, Department of Psychiatry, University of Colorado School of Medicine

## Abstract

Adolescence is characterized by ongoing neurodevelopment and psychosocial development, resulting in a unique window to the adverse effects of traumatic events and substance use. In addition, trauma- and stressor-related disorders and substance use disorders (SUDs) commonly co-occur in adolescents. Youth with interpersonal violence and who have experienced multiple past traumas, or poly-victimization, are at the highest risk of developing these co-occurring disorders. There is a strong bidirectional relationship between traumatic events and substance use that predisposes youth to developing post-traumatic stress symptoms (PTSS) and SUDs. PTSD and states of substance intoxication and withdrawal also exhibit overlap in symptomatology. High rates of comorbidity may be explained in part by the self-medication hypothesis, that posits that individuals use substances to temporarily alleviate trauma-related symptoms. However, this results in negative reinforcement, often with increasing patterns of substance use and worsening symptoms of hyperarousal, dysphoria, and anxiety. In addition, PTSS and substance use problems share common risk factors and neurobiologic etiology, conceptualized as the susceptibility hypothesis. Youth who experience traumatic events and/or have substance use problems access the healthcare system at multiple levels, including through acute care and crisis services. Notably, substance use in adolescence increases the likelihood of experiencing a traumatic event, and youth presenting to the emergency department for substance-related problems are at higher risk of having a PTSD. Youth presenting for mental health, behavioral, or substance-related problems should be screened for PTSS and substance use problems. Given the strong clinical overlap and bidirectional relationship, evidence-based treatment integrates management of both disorders. An interdisciplinary approach with psychotherapy, psychopharmacologic therapy, and case management is often vital to engaging and maintaining youth in treatment.

## Introduction

Potentially traumatic events, including adverse childhood events (e.g., maltreatment, caregiver psychopathology, violence in the home), are common among European children and adolescents with data from multi-country surveys showing that approximately 50% of youth have experienced at least one adverse childhood event in their lifetime^1,2^. Exposure to traumatic events during childhood and adolescence is associated with a myriad of mental health concerns including posttraumatic stress symptoms (PTSS) and posttraumatic stress disorder (PTSD) and substance use disorders (SUD). Moreover, there is evidence of reciprocally reinforcing influences of trauma and substance use, such that earlier and more frequent experiences in one domain predict greater risk for problems in both domains over time.

PTSD is generally characterized by persistent intrusive thoughts associated with a traumatic event, negative alterations in mood and cognitions, avoidance of reminders of the traumatic event, and hyperarousal in response to internal or external cues that trigger traumatic stress reactions^3-5^. Epidemiological studies estimating population prevalence of traumatic stress disorders are rare, particularly outside of the United States. One study of Swiss adolescents found that 56% of youth experienced a potentially traumatic event with 4.2% of youth currently meeting criteria for post-traumatic stress disorder^6^. These estimates are consistent with those found in the United Kingdom^7^. However, studies likely underestimate the experiences of youth living in areas with active war or refugee youth who were displaced due to war because of the the challenges of conducting research in those settings^8^.

Estimates of substance use prevalence among European youth are more widely available. Data from the 2019 European School Survey Project on Alcohol and other Drugs showed an average of 79% of lifetime use of alcohol among 15- to 16- year-olds across 49 European countries, with 47% of youth using alcohol in the past 30-days^9^. On average 16% of youth engaged in lifetime cannabis use across 49 countries, while 5% of youth have used other illicit substances such as ecstasy, cocaine, hallucinogens, heroin, or gamma hydroxybutyrate (GHB). However, the prevalence of substance use varies across countries. Cannabis and illicit drug use among youth appear lowest among Nordic countries (e.g., Sweden and Norway) and Southeast Europe (e.g., Greece and Kosovo) with lifetime prevalence lower than 10%, and higher among Baltic Countries (e.g., Lativa, Lithuania, and Estonia) and Central European Countries (e.g., Germany, Poland, and Czechia) with lifetime prevalence higher than 20%. Similar patterns of alcohol and cannabis use have also been found in the Health Behaviour in School-aged Children (HBSC) survey^10^.

A SUD is characterized by continuing to use substances despite substance-related problems, broadly categorized as impaired control, social impairment, risky use, and pharmacological effects (e.g., tolerance and withdrawal)^11^. Data from the 2021 Global Burden of Disease study estimated that 3.13% of 15- to 19-year-old European youth are living with a SUD^12^. Approximately 1.59% of youth have cannabis use disorder, 1.10% of youth have alcohol use disorder, and less than 1% of youth have another illicit SUD. The prevalence of SUDs also varied across European countries with 4.5% of youth in Western Europe having a SUD, followed by 2.60% of youth in Eastern Europe, and 2.45% of youth in Central Europe.

While estimates of SUD are relatively low, trauma-related and substance-related problems commonly co-occur in youth, resulting in disproportionately high rates of SUDs among youth with PTSS or PTSD– and vice versa – relative to population prevalence. Approximately 24-30% of youth with PTSD also meet criteria for a comorbid SUD, whereas 13-25% of youth with a primary SUD have a diagnosis of PTSD.^13^ Estimates of co-occurring disorders are even higher among adults (30-50%), with up to 85% of individuals seeking treatment for PTSD having a comorbid SUD^13,14^. When considering the discrepancy between the prevalences of co-occurring PTSD and SUD in youth and adults, it is important to remember that traumatic exposures are often underestimated and difficult to verify in youth. Many youth do not have insight into the relationship between traumatic exposures and trauma-related mental health problems^13^. Youth may also have significant PTSS without meeting criteria for PTSD.

Overall, trauma- and substance-related disorders are likely to be underdiagnosed in youth, perpetuating challenges accessing evidence-based care and often prolonging trauma- and substance-related problems into adulthood. Herein, we will explore the strong bidirectional relationship between trauma and substance use, review the clinical implications of comorbid trauma- and substance-related disorders, discuss evidence-based treatment of these co-occurring disorders, and propose recommendations for emergency medicine providers.

## Bidirectional relationship

There is a well-established bidirectional connection between potentially traumatic events, trauma- and stressor-related disorders, and substance use^13,15-17^. Traumatic events during childhood increase youths' risk for substance use problems, including higher risk of developing a SUD^18-22^. The type of traumatic event and experiencing multiple traumatic events, or poly-victimization, may also contribute significantly to the increased risk of substance use problems. Of potentially traumatic events, interpersonal violence (e.g., physical or sexual assault) may lead to the highest risk of substance use problems^15,16^. In fact, youth who are physically or sexually abused have a 12-fold higher odds of regularly using cannabis or alcohol by age 10 years and an eight-fold higher odds of heavy drinking by age 14 years^23^. Similarly, exposure to multiple traumatic experiences is associated with a three to five times higher odds of developing a SUD compared to youth who have experienced a single traumatic event^16,24^. Similarly, substance use during adolescence is associated with increased risk of experiencing a potentially traumatic event^25^. Adolescent substance use is associated with higher levels of risky behaviors and victimization^26^.

A diagnosis of PTSD is also associated with increased risk of substance-related problems. Adolescents with PTSD are four times more likely to have alcohol use disorder, six times more likely to have cannabis use disorder, and nine times more likely to have a SUD for other illicit drugs (e.g., cocaine, methamphetamines, opioids) than adolescents without PTSD^27^. Among youth presenting to the emergency department, a PTSD diagnosis is associated with higher odds of being diagnosed with a SUD (aOR = 1.36, 95 %CI:[1.04, 1.79])^28^. A diagnosis of PTSD in youth is also associated with increased risk of using multiple substances, or polysubstance use^29^. Factors that may explain the high rates of comorbidity between trauma- and substance-related disorders have been outlined in the shared vulnerability, susceptibility, and self-medication hypotheses (Table 1), as well as in models detailing common neurobiologic etiology between the disorders.

### SHARED VULNERABILITY, SUSCEPTIBILITY, AND SELF-MEDICATION HYPOTHESES

While many youths experience potentially traumatic events, the majority do not develop PTSD. Similarly, most youths who use psychoactive substances ultimately do not develop a SUD. However, the co-occurrence of trauma-related and substance-related problems sharply increases the prevalences of both PTSD and SUD. This suggests a collective vulnerability that predisposes individuals to developing these comorbid disorders^30^. The shared vulnerability hypothesis proposes that trauma- and substance-related disorders share common risk factors^30,31^. These risk factors include individual psychiatric (e.g., impulsivity) and biologic (e.g., family history of substance use and intergenerational trauma) factors, social and familial factors (e.g., inadequate parental support), and other environmental factors (e.g., availability of substances).

Overall, among those who experience traumatic events, youth with a SUD are at up to four times higher risk of developing PTSD^32^ and more likely to experience prolonged or exacerbated PTSS^33^. First proposed by Chilcoat and Breslau^34^, the susceptibility hypothesis posits that when individuals experience a traumatic event, those who use substances are at higher risk of developing PTSD than those who do not^13,14,34,35^. Substance use disrupts the biologic stress response system, interfering with the youth's ability to effectively cope with traumatic events^32^. This results in increased anxiety, hyperarousal, and poor coping following a traumatic event^30^. Further, subsequent use of substances to avoid trauma-related symptoms may prevent habituation of traumatic memories^36^. Taken together, these findings strongly support that youth who misuse substances are more likely to develop trauma-related problems after experiencing a traumatic event.

When considering the relationship between comorbid trauma- and substance-related disorders, PTSS have been demonstrated to mediate the relationship between traumatic events and SUDs in multiple studies^27,37-39^. Implicit in the name, the self-medication hypothesis postulates that some individuals with PTSS are more prone to use substances to cope with negative internal experiences^33^. Substance use becomes a mechanism to avoid intrusive symptoms, alleviate hypervigilance, and seek relief from negative affective states^33,40-42^. In turn, temporary relief from PTSS positively reinforces substance use^43^, increasing the likelihood of progression to a SUD. While the self-medication hypothesis is widely accepted, relatively little rigorous research has been conducted to evaluate the longitudinal relationship between reported self-medication and trauma- and substance-related problems, especially in adolescents^33^.

### COMMON NEUROBIOLOGIC ETIOLOGY

Adolescence is a vital period of neurodevelopment that is uniquely vulnerable to the harms of potentially traumatic events and substance use. Experiencing a traumatic event and misusing substances both dysregulate the biologic stress response system and result in neurobiologic changes^13,44^. Accumulating research shows that trauma- and substance-related neurobiologic changes affect emotional regulation, motivation, and physiologic stress response^13,44,45^. Synthesis of the literature suggests changes in the mesolimbic dopamine system, noradrenergic system, hypothalamic-pituitary-adrenal (HPA) axis, amygdala, and ventromedial prefrontal cortex (PFC) moderate arousal, hypervigilance, and anxiety, traits central to PTSD and drug-seeking behaviors in SUD^13,46^.

The mesolimbic dopamine system mediates reward-motivated behaviors that underly SUD, including reward learning and cognitive processes fundamental to decision making^47^. Downregulation of mesolimbic dopaminergic signaling in individuals who chronically use psychoactive substances also results in transition from using for positive effects (e.g., euphoria) to using to prevent negative effects (e.g., withdrawal, anxiety, hyperarousal, and depression)^13^. Ultimately, the transition from positive effects to negative effects subsequently reinforces compulsive patterns of intoxication and withdrawal, which subsequently worsens both trauma- and substance-related problems in youth. For example, dopaminergic-related changes include aberrant learning, reward deficiency, and anhedonia, which subsequently leads to drug craving and a higher risk of relapse in response to stressors^48^.

Changes in mesolimbic dopamine signaling that are common between PTSS and SUDs are in part mediated by the noradrenergic system, a constellation of several nuclei that synthesize and release the catecholamine norepinephrine^49^. The locus coeruleus, which projects throughout the cerebral cortex and thalamus, regulates several cognitive functions central to stress response, including arousal, attention, emotional regulation, learning, motor control, and cardiopulmonary functions^50,51^. Traumatic stress activates the locus coeruleus, which then mediates the “fight-or-flight” response via the sympathetic nervous system^13,44^. Trauma-mediated changes in noradrenergic signaling may also result in dysfunctional cue reactivity that reinforces motivation to misuse substances. Further, alterations in noradrenergic signaling and sympathetic tone associated with opioid withdrawal and alcohol use also cause dysregulated response to traumatic stress^13^.

Noradrenergic signaling also results in stimulation of the HPA axis^13^. The HPA axis consists of signaling pathways that mediate physiologic response to stress^52^. Multiple substances alter HPA and noradrenergic signaling, including alcohol, cannabis, cocaine, opioids, and nicotine^53^. Norepinephrine is a neurotransmitter that stimulates hypersecretion of corticotropin releasing hormone (CRH) in the hypothalamus^13^. CRH then stimulates the pituitary gland to secrete adrenocorticotropic hormone (ACTH), which in turn causes the adrenal gland to produce epinephrine and cortisol^54^. Traumatic stress and substance use both alter HPA-mediated signaling, resulting in a negative feedback loop that leads to hypersensitivity to stress and a maladaptive stress response^13^. As with mesolimbic dopaminergic and noradrenergic pathways, hyperarousal is characteristic of trauma- or substance-mediated changes in HPA axis signaling^13^.

The mesolimbic dopamine system, noradrenergic system, and HPA axis converge within the amygdala, a part of the limbic system that is central to processing emotions such as fear and anxiety as well as motivation^55^. CRH levels in the amygdala are positively correlated with hyperarousal and increased fear in response to stress^56^. In addition, hyperactivity of the amygdala, which is induced by both PTSS and alcohol use^57^, is one of the most consistent neuroimaging findings observed in PTSD^58^. It is postulated that this hyperactivity subsequently underlies hyperarousal and fear conditioning in PTSD and drug-seeking behaviors in response to a cue or stressor in SUD^13^.

The amygdala receives inputs from multiple cortical regions, including the PFC. Hypoactivity in the ventromedial PFC, which has been reported in both PTSD and SUD, results in amygdala hyperactivity^13^. In youth, ventromedial PFC hypoactivity is also correlated with the duration of PTSD symptoms and is hypothesized to negative affective symptoms, anxiety, and regressive behavior^58,59^. Among individuals with alcohol use disorder, hypoactivity of the PFC is associated with craving and binge drinking^13,57^. Hypoactivity is also associated with higher rates of relapse in response to stress^57^, implicating changes in the PFC in maladaptive stress response. Overall, while neuroimaging studies are limited by sample size and methodology, synthesis of the literature consistently points to amygdala hyperactivity and PFC hypoactivity, as well as PFC-amygdala connectivity, as central to maladaptive stress response in PTSD and SUD^13^.

## Clinical implications

The co-occurrence of trauma- and substance-related disorders leads to several clinical challenges. As discussed above, common neurobiologic etiology and overlapping symptomatology reinforces maladaptive stress response behaviors including substance misuse, which results in poorer behavioral health outcomes. In a large sample of adolescents receiving treatment for SUD (n=20,069), greater PTSS was found to predict increased risk of return to substance use and more substance use at treatment completion was associated with greater PTSS^60^. Further, past traumatic experiences are associated with poorer treatment outcomes in youth entering treatment for SUDs^61^. Unremitted PTSS is also associated with poorer substance use treatment outcomes compared with individuals for whom PTSS has remitted^62^. Individuals with alcohol use disorder experience a greater number of reexperiencing symptoms, which may mediate difference in treatment outcomes.^62^ Similarly, individuals with PTSD who have a comorbid SUD experience significantly poorer physical and mental health and greater disability than those without a SUD^63^.

Of particular concern, traumatic events and trauma- and stressor-related disorders put adolescents at increased suicide risk^16,64,65^. Similarly, substance use is an independent risk factor for suicidal behavior, including increased risk of suicide attempts^66^. As with the risk of substance use, interpersonal violence, particularly sexual violence, is associated with markedly higher odds of experiencing suicidal ideation or attempting suicide^67^. Increased risk of suicide among youth with comorbid trauma and SUDs may be mediated by substance use, or impulsivity and risk taking while intoxicated, particularly among sexual and gender minority youth^68^.

## Evidence-based treatment

When considering the relationship between comorbid trauma- and substance-related disorders, PTSS have been demonstrated to mediate the relationship between traumatic events and SUDs in multiple studies^27,37-39^. Despite the common co-occurrence of traumatic stress and substance use, few interventions have been developed and tested for that specific comorbidity^69,70^. Several effective treatments have been shown to be safe and effective in treating concerns in each clinical domain. Most evidence-based treatments for trauma-related mental health concerns and substance use in adolescents involve time-limited courses of outpatient care where families work with a behavioral health clinician to address their needs^71-73^. While these treatments are unlikely to be provided in an emergency medicine setting, familiarity with these models can help guide discharge planning, referral and case management supports, and coordination with families and other healthcare providers.

### PTSD TREATMENTS

With regard to PTSD, trauma-focused cognitive behavioral therapy (TF-CBT)^74^ has the most robust research evidence supporting its efficacy in reducing PTSD symptoms, including among youth who are using substances. TF-CBT has been evaluated in over two dozen randomized controlled trials (RCTs), including in Europe^75,76^. TF-CBT is implemented in phases. First, youth and caregivers are equipped with education about trauma and common reactions to trauma, as well as strategies for managing distress. A key feature of TF-CBT is the use of exposure to trauma-related thoughts and feelings throughout treatment to help youth and families overcome avoidance and manage other behavioral health symptoms. This gradual exposure begins early in TF-CBT and is the focus of the second phase, which includes trauma narration and processing. Finally, youth and caregivers come together in conjoint sessions to foster open communication about the trauma and related issues and to set plans for promoting safety, resilience, and healthy future development. Reviews of TF-CBT support its effectiveness in reducing PTSS, depression, grief, and other trauma-related concerns^73,75^. Evidence suggests TF-CBT is also effective in treating PTSS in youth with complex clinical profiles^77^, including those who report past or current substance use^78^. Other empirically supported treatments that have been found effective in addressing traumatic stress symptoms include eye movement desensitization and reprocessing (EMDR), cognitive processing therapy (CPT), and prolonged exposure (PE) adapted for adolescents^79,80^.

### SUD TREATMENTS

There are also several established evidence-based treatments for SUDs in youth^71,72^. These interventions are also typically rooted in cognitive-behavioral principles, whereby adolescents and caregivers are taught skills for identifying and managing SUD symptoms and antecedents and consequences of substance use. Some models focus largely on working with the adolescent, whereas others focus more directly on caregivers and the family system. Effective individual therapies include CBT, motivational interviewing/ motivational enhancement therapy (MI/MET), contingency management (CM), and combinations of these interventions. Adolescent-focused models typically involve guiding youth toward personalized treatment goals through manualized sessions focused on topics like emotion regulation skills, assertive communication (e.g., refusal skills), problem-solving, and cognitive processing. Family-based SUD treatments include brief strategic family therapy (BSFT), functional family therapy (FFT), multidimensional family therapy (MDFT), and multisystemic therapy (MST). These models place substantial emphasis on the roles caregivers play in shaping their children's environments, including through intentional applications of rewards and other consequences, to modulate the risk of substance use. Collectively, these treatments have been shown to reduce adolescent substance use frequency and quantity, as well as substance-related problems.

### INTEGRATED TREATMENTS

Given overlap between components of PTSD and SUD treatments for adolescents (e.g., coping skills training; overcoming avoidance; caregiver involvement; etc.), there has been a long-standing call for manualized, integrated treatments designed to address comorbid PTSD and SUD in a single course of care^81^. Becker and colleagues (2023) recently performed a systematic review and identified 10 RCTs evaluating interventions designed to address substance use and comorbidities in an integrated fashion^69^. In line with the broader literature on SUD and PTSD interventions, the identified treatments involved cognitive-behavioral principles that were typically implemented at the individual youth or family level. Nearly all identified studies were conducted in the U.S., and only two were rated as strong using Effective Public Health Practice Project (EPHPP) Quality Assessment Tool. The first study, Stepped Collaborative Care, was not found to outperform treatment as usual for PTSD or SUD^82^.

The second study with a strong EPHPP rating was an RCT evaluating Risk Reduction through Family Therapy (RRFT)^78^. RRFT was also the only treatment model focused on PTSD identified in a recent systematic review of evidence-based integrated treatments for psychiatric disorders among youth who use cannabis^70^. Guided by a theoretical model that emphasizes reducing risk factors and boosting protective factors across multiple levels of the adolescent's ecology, RRFT incorporates elements of CBT and family therapy for PTSD with SUD. Early studies supported the feasibility, acceptability and initial efficacy of RRFT in open and pilot randomized trials^83,84^. RRFT was evaluated in a large RCT compared to TF-CBT, the "gold standard" for PTSD, in a sample of 124 adolescents^78^. While both conditions resulted in improved PTSD and substance use outcomes, youth assigned to the RRFT condition showed greater reductions in number of days of substance use, PTSD avoidance symptoms, and other outcomes up to 18 months after enrollment. An additional trial of RRFT is currently underway to evaluate its effects relative to a comprehensive adolescent SUD treatment^85^.

### PHARMACOLOGICAL INTERVENTIONS

While psychotherapy is first-line for PTSS and PTSD in youth, pharmacologic treatments may also be utilized in conjunction with trauma-focused therapy^86^. To date, clinical trials have found little evidence for medications as monotherapies for PTSS. The most prescribed medications for PTSS are selective serotonin reuptake inhibitors (SSRIs). Randomized controlled trials (RCTs) have demonstrated the safety and tolerability of sertraline and citalopram in youth with PTSS^86^. By extension, other SSRIs including fluoxetine and escitalopram are also prescribed in youth with PTSS off-label. Of note, clinical trials have found limited evidence of between group differences in youth who receive an SSRI vs placebo, which may be due to both groups receiving psychotherapy, such as TF-CBT^86^. Utilization of an SSRI or other antidepressant should also be considered in youth with comorbid PTSS and depression or anxiety.

Other common targets of pharmacotherapy in PTSS and PTSD are sleep/nightmares and hyperarousal. To date, antiadrenergic agents such as clonidine, a central alpha-2 adrenergic partial agonist, and prazosin, an alpha-1 adrenergic antagonist, have not been rigorously studied in youth with trauma. Antiadrenergic agents may function primarily by dampening catecholamine transmission, which lowers sympathetic nervous system tone in youth with PTSS^44^. In addition, case reports and small open-label trials support the use of these medications for sleep and hyperarousal in combination with psychotherapeutic interventions^86^. Propranolol, a beta-adrenergic antagonist, may also be used off-label for hyperarousal and comorbid anxiety^44,86^.

As with trauma-related disorders, psychotherapy is the primary intervention for youth with SUDs and medications may be used as an adjunct to alleviate symptoms, reduce cravings, and prevent relapse^87^. Pharmacotherapies may generally be considered as agonist therapy (e.g., nicotine replacement products, methadone, and buprenorphine), adjunct treatments for symptomatic relief of withdrawal or cravings (e.g., clonidine, gabapentin, and acamprosate), and other medications (e.g., disulfiram). However, in youth there is a paucity of research studying the efficacy of medications in SUDs. As a result, most medications used for SUDs in youth are off-label and may not be widely available.

## Recommendations for emergency medicine providers

Too many youths with co-occurring trauma- and substance-related disorders do not receive evidence-based treatment. Often, these youths access the healthcare system through acute care settings in times of crisis. There are several ways emergency medicine providers can support the needs of adolescents with co-occurring traumatic stress and substance use.

First, it is important to recognize that trauma and substance use are often intertwined. Understanding these connections can increase sensitivity when counseling youth with trauma who present to the emergency department for substance-related problems. It also informs the broader set of clinical needs these families often face beyond the acute presenting problem. Effective care for this population often requires coordination and collaboration among multidisciplinary team members including physicians, nurses, social workers, care managers, and other allied health professionals and youth-serving systems.

Second, systematic, universal screening of adolescents for trauma exposure and substance use should be implemented in acute care settings. Note that while youth may experience acute stress in relation to the event that resulted in the emergency department visit, they may also endorse trauma-related symptoms pertaining to prior trauma and adversity. Several brief screening tools have been validated in youth and can be implemented efficiently in acute care settings. Some of these tools are also available in numerous languages, increasing their practical and clinical utility in diverse settings and populations^8,88-93^. Use of universal screening procedures can help minimize bias in case identification and help ensure at-risk youth are offered appropriate services. While youth may be reluctant to disclose sensitive information in the context of an emergency department visit, implementing standardized, trauma-informed screening protocols in a private, confidential setting will improve valid responding.

Third, when adolescents screen positive for trauma and/or substance use, a more thorough evaluation of trauma- and substance-related disorders should be conducted to better understand current functioning and to guide case management and treatment needs. Such evaluations should include diagnostic assessments, as well as assessment of related psychosocial factors like protective factors that may be leveraged to promote resilience. When available, psychiatric services integrated within the emergency department (e.g., psychiatric emergency services or consult-liaison service) may be utilized for further diagnostic evaluation and disposition recommendations. In situations where it is not feasible to conduct an evaluation during the emergency department encounter, a referral to a community-based assessment provider should be included in the discharge plan.

Fourth, adolescents with co-occurring trauma- and substance-related disorders should receive referrals to effective community-based treatment services. Whenever possible, this should include integrated treatments that address comorbid disorders. In cases where such services are not locally available, referrals to evidence-based treatment for PTSS and/or SUD may be made as these treatments can still be effective among youth with complex, comorbid clinical needs. Where integrated treatments are not readily available, emergency medicine providers can center advocacy for building a network of treatment resources on the clinical needs and experiences of these youth and their families.

Fifth, providers should implement brief motivational interventions to help adolescents and families experiencing trauma-related and substance-related problems connect to appropriate follow-up services^94^. When youths are given a handout with community treatment resources, treatment engagement rates are low. Although delivery of comprehensive treatments for PTSS and SUD are often beyond the scope of what can be offered in acute care settings, motivational interventions may increase post-hospital treatment engagement. Brief interventions can also help reduce substance use and future victimization^94,95^. Such interventions often require 5-15 minutes and can be delivered by physicians, nurses, social workers, case managers, or other members of the healthcare team. ED teams can also offer psychoeducation and anticipatory guidance about trauma recovery and when adolescents and families should seek professional support as they recover from an injury or illness. Lastly, ED personnel should follow up with patients and their families to monitor their efforts to obtain treatment.

## Conclusion:

In conclusion, healthcare professionals — especially those working in emergency departments and other acute care settings — often play a key role in identifying and responding to trauma, traumatic stress, and substance use in adolescents. These clinical concerns often co-occur, which can complicate recovery of trauma- and substance-related disorders. Understanding the epidemiology, etiology, and evidence-based intervention strategies can equip clinicians with the knowledge and tools they need to care for these youths and their families.

## Figures and Tables

**Table 1:** Hypotheses to explore the co-occurrence of PTSD and SUD

Hypothesis	Description	Clinical implications
Shared vulnerability	PTSD and SUD share common risk factors at the individual, social and familial, and environmental levels that predispose individuals to develop these disorders.	Targeting universal and selective interventions to mitigate risk factors and enhance protective factors will decrease the risk of developing PTSD and SUD in at-risk youth.
Susceptibility	Substance use results in dysregulation of the biologic stress response system, which increases the risk of developing trauma-related problems including anxiety hyperarousal due to dysregulation of the biologic stress response system.	Youth who are misusing substances and experience a traumatic event are more likely to develop trauma-related problems. It is important to discuss harm reduction strategies to avoid traumatic exposures and to increase support immediately following traumatic exposures.
Self-medication	Individuals who have experienced trauma are prone to misuse substances to avoid or temporarily alleviate trauma-related problems, which subsequently reinforces substance misuse and increases the risk of developing PTSD and SUD.	Youth with trauma-related problems struggle abstain from substances because of perceived benefits of substance misuse. States of intoxication and withdrawal also mimic trauma-related symptoms, and over time, cycles of intoxication and withdrawal worsen both trauma- and substance-related problems. It is essential to adequately manage substance withdrawal and address cravings and risk of relapse in youth with PTSD who misuse substances.

**Table 2:** Treatments for PTSD, SUD, and co-occurring PTSD and SUD in adolescents with strong empirical support

PTSD treatments	SUD treatments	Integrated PTSD+SUD treatments
• Trauma-focused cognitive behavioral therapy (TF-CBT) • Eye movement desensitization and reprocessing (EMDR) • Cognitive processing therapy for adolescents (CPT) • Prolonged exposure for adolescents (PE)	• Cognitive behavioral therapy (CBT) • Motivational interviewing (MI) integrated with CBT (MI+CBT) • Contingency management (CM) • Family-based therapies o Brief strategic family therapy (BSFT) o Functional family therapy (FFT) o Multidimensional family therapy (MDFT) o Multisystemic therapy (MST)	• Risk reduction through family therapy (RRFT)

**Table 3:** Recommendations for emergency medicine providers

1.Understand and recognize etiological and functional connections between trauma exposure, traumatic stress, and substance use 2.Screen routinely for trauma exposure, traumatic stress, and substance use using standardized, developmentally appropriate instruments and protocols that gather data from both adolescents and caregivers in private settings 3.Conduct diagnostic and psychosocial evaluations among adolescents who screen positive for trauma or substance use to better understand risk and resilience factors underlying symptoms and impairment, as well as to guide treatment planning 4.Refer adolescents and families in need of PTSD and/or SUD treatment services to behavioral health providers who offer evidence-supported services – including integrated treatments for co-occurring disorders, when available – as well as case management resources when needed 5.Implement brief motivational interventions to help adolescents and families experiencing trauma-related and substance-related problems connect to appropriate follow-up services
